# The Probiotic *Bacillus hwajinpoensis* Colonizes the Digestive System of *Crassostrea gigas* Larvae and Protects Them from *Vibrio alginolyticus* Infection

**DOI:** 10.3390/microorganisms11122918

**Published:** 2023-12-04

**Authors:** Yu-Dong Zheng, Bo-Wen Huang, Xiang Zhang, Chen-Feng Liu, Lu-Sheng Xin, Chong-Ming Wang, Chang-Ming Bai

**Affiliations:** 1 State Key Laboratory of Mariculture Biobreeding and Sustainable Goods, Yellow Sea Fisheries Research Institute, Chinese Academy of Fishery Sciences, Qingdao 266071, China; zhengyudong032@163.com (Y.-D.Z.); huangbw@ysfri.ac.cn (B.-W.H.); zhxiang1997@126.com (X.Z.); liucf9864@163.com (C.-F.L.); xinls@ysfri.ac.cn (L.-S.X.); wangcm@ysfri.ac.cn (C.-M.W.); 2 Laboratory for Marine Fisheries Science and Food Production Processes, Qingdao National Laboratory for Marine Science and Technology, Qingdao 266237, China; 3 Key Laboratory of Maricultural Organism Disease Control, Qingdao Key Laboratory of Mariculture Epidemiology and Biosecurity, Ministry of Agriculture, Qingdao 266071, China

**Keywords:** *Bacillus hwajinpoensis*, probiotic, *Vibrio alginolyticus*, pathogenicity, *Crassostrea gigas*

## Abstract

The Pacific oyster *Crassostrea gigas* is one of the most important cultured marine species around the world. Production of Pacific oysters in China has depended primarily on hatchery produced seeds since 2016, with the successful introduction and development of triploid oysters. However, the seed supply of Pacific oysters is threatened by recurring mass mortality events in recent years. Vibriosis is the most commonly encountered disease associated with intensive oyster culture in hatcheries and nurseries. *Vibrio alginolyticus* and *Bacillus hwajinpoensis* were the two strains with pathogenic and probiotic effects, respectively, identified during the Pacific oyster larvae production. To monitor their colonization process in Pacific oyster larvae, green fluorescent protein (GFP) and red fluorescent protein (RFP) were labeled to the pathogenic *V. alginolyticus* and the probiotic *B. hwajinpoensis* stain, respectively. The pathogenic and probiotic effects of the two strains during the colonization process were then assessed. Stabile expression of GFP and RFP were observed in corresponding stains, and the capabilities of growth, biofilm formation and in vitro adhesion of GFP- and RFP- tagged stains were not significantly different from those of the wild-type strains. Usage of probiotics of 10^5^ CFU/mL significantly inhibited the growth of pathogenic *V. alginolyticus* and reduced the mortality of D-sharped larvae. Both the pathogenic and probiotic strains employed a similar route to enter and colonize the oyster larvae, which indicates that competing with pathogens for binding and spreading sites were one of the mechanisms of *B. hwajinpoensis* to provide the probiotic effects to oyster larvae. In summary, employment of fluorescence-tagged pathogenic and probiotic strains simultaneously provides us with an excellent bioassay model to investigate the potential mechanisms of probiotics.

## 1. Introduction

The Pacific oyster *Crassostrea gigas*, also referred to as *Magallana gigas*, is a bivalve mollusk originally distributed in Japan, China, the Korean Peninsula, and Sakhalin Island [1]. As the potential for rapid growth and tolerance of a wide range of environmental conditions, the species has been introduced for cultivation in many coastal areas of North and South America, Europe, Australia and East Asia [2]. The production of Pacific oysters has increased rapidly in recent years in China, and reached 1.46 million tons in 2018 [3]. The Pacific oyster aquaculture relied on diploid seeds before 2016 in China, which were primarily collected from local waters where oyster aquaculture is practiced [4]. In recent years, the triploid oysters were successfully developed in China, and widely embraced by farmers [4]. It is estimated that the production of triploid oysters has accounted for over 70 percent of the production in China [5]. Because the triploid pacific oyster is generally sterile, the seed supply depends completely on hatchery production. To meet the huge demands for triploid oyster seeds, the bivalve hatcheries have expanded rapidly, which has brought new threats to the industry [6]. This is due to, as all researchers and practitioners have noted, more frequent occurrences of production failure of oyster seeds [4]. Mass hatchery crashes have caused shortages in seed oysters for commercial shellfish producers. Epidemiological investigation of hatchery epidemics indicated that Ostreid herpesvirus 1 (OsHV-1), *Vibrio algniolyticus*, *Vibrio mediterranei*, and *Pseudoalteromonas* spp. were the potential pathogenic microorganisms [6,7,8,9]. The poor facilities to eliminate pathogens from seawater, poor biosecurity concepts during the operation, and management were assumed to be responsible for the insufficiency of disease prevention [6,10].

Hatchery production of bivalves constitutes several activities that are highly susceptible to bacterial contamination, which includes introduction of broodstocks, phytoplankton, and seawater to the cultivation system [11]. This is because microorganisms, including pathogenic *Vibrio* spp., were natural component of coastal waters, which makes them difficult to avoid [12]. Vibriosis disease, caused by various Vibrio species, is reported as the most common disease in association with mass mortality in bivalve hatcheries [13]. Bacillary necrosis associated with *Vibrio tubiashii* infection (reclassified as *Vibrio coralliilyticus* latterly [14]) has been responsible for major hatchery crashes of oysters since 1965 [15,16,17]. According to the summary of Dubert et al. (2017), 12 Vibrio species of 6 clades have been identified as the etiological agent responsible for the larval and spat mortalities of different hatchery cultured bivalve species worldwide. The antibiotics have been applied to kill or inhibit pathogenic Vibrio growth [13,15]. While the overall effect of antibiotics varies considerably among cases, antibiotic usage is discouraged because of bacteria’s potential development of resistance and negative impacts on healthy microbiota [18,19].

Probiotics are live microorganisms that are intended to have health benefits to the host when consumed or applied in adequate amounts [20]. The beneficial effects of probiotics have not only been linked to human health, but also widely recognized by livestock and aquaculture production [21]. Candidate probiotics from over 17 genera have been developed for use during the early stages of bivalve development in hatchery and nursery [22,23,24]. Several studies investigated certain mechanisms behind the beneficial effects, such as pathogen inhibition, secretion of antimicrobials, immunomodulation, host growth promotion, competition for nutrients with pathogens, and improvement of water quality [22,23,25]. For example, *Phaeobacter inhibens* S4 (S4) and *Bacillus pumilus* RI06–95 (RI) protect larval Eastern oysters (*Crassostrea virginica*) from *Vibrio coralliilyticus* RE22 infection through different mechanisms [19,26]. The health benefits of S4 were derived from biofilm formation, secretion of the antibiotic tropodithietic acid, quorum quenching, and host immune modulation [25,27,28]. Mechanisms of RI include stimulating the immunity and promoting enhanced digestion in oysters [26,28]. Vibrio strain OY15 improved the survival of Eastern larval oysters by immuno-stimulation of oyster hemocytes [29,30].

The pathognomonic signs and infection process of Vibriosis in bivalve larvae were firstly investigated by means of histological observation [15]. Subsequently, immunohistochemical staining was employed to detected the colonization sites in great scallop (*Pecten maximus*) larvae [31]. But histological and immunohistochemical staining approaches could not trace the colonization process of specific bacteria [31]. Itis difficult to estimate the distribution and loads of pathogens at different time-points post infection. Fluorescent proteins allow direct observation of molecular processes in living systems and are therefore widely used in biochemistry, cell biology, and microbiology [32]. The main fluorescent protein currently used is green fluorescent protein (GFP), and the labelling method is well established and widely used [33]. GFP was firstly used to trace the colonization process of bacteria in bivalve larvae of Manila clam (*Ruditapes philippinarum*) [34], then Chilean scallop (*Argopecten purpuratus*) [35], and blue mussel (*Mytilus edulis*) [36]. The results demonstrated that the microvilli and cilia of the epithelial cells of the gastrointestinal tract were the first to be destroyed by Vibrio and these sites were the most sensitive to Vibriosis [36]. The colonization routes of bacteria in these larvae could be defined to three stages [34,36]. At the first stage, the bacteria were filtrated through the vellum, and entered the stomach through the esophagus. Then, the bacteria diffused to the surrounding organs localized in the dorsal and ventral regions, and finally, the whole visceral mass [34]. Pathological signs characterized by necrosis of digestive organs and disorganized cilia were correspondingly observed along the colonization process [13]. However, the processes of bacterial colonization and infection in oyster larvae have not been investigated using the labeling method according to our knowledge.

Previously, we isolated a strain of *Bacillus hwajinpoensis* from healthy Pacific oyster larvae, which showed typical features of potential probiotic candidates. It exhibited anti-Vibrio activity and increased oyster larvae survival when challenged with *V. algniolyticus*. However, the mechanisms by which the probiotic *B. hwajinpoensis* enters the oyster, spreads among individuals, or interacts with the pathogenic *V. algniolyticus* remain thus far almost unknown. In this study, we tagged the probiotic *B. hwajinpoensis* with red fluorescent protein (RFP) and the pathogen *V. algniolyticus* with green fluorescent protein (GFP). Subsequently, we investigated the colonization process of these strains in Pacific oyster larvae. Finally, we assessed the bacterial inhibition effects and probiotic effects of *B. hwajinpoensis* on the larvae.

## 2. Materials and Methods

### 2.1. Bacterial Strains and Culture Conditions

The pathogen, *V. alginolyticus* CD, was isolated from the diseased *C. gigas* larvae, and its pathogenicity had already been verified in a previous study [6]. The probiotics, *B. hwajinpoensis* RC, were selected from healthy *C. gigas* larvae, exhibiting anti-Vibrio activity and beneficial effects on the survival of oyster larvae [6]. Both strains used in this study were cryopreserved isolates stored at −80 °C. To facilitate recovery, the strains were inoculated in 2216E medium containing 5 g/L tryptone, 1 g/L yeast extract, and 0.01 g/L FePO_4_. The inoculation process took place at 28 °C.

### 2.2. Construction of Plasmids, GFP/RFP-Tagged Strains

The plasmid preparation, extraction of DNA fragments from agarose gels, and purification of PCR products were performed according to the manufacturer’s instructions of the relevant kits (Tiangen Biotech, Beijing, China).

The green fluorescent protein (GFP) and the Turbo red fluorescent protein (RFP) with gene lengths of 720 bp and 696 bp, respectively, were employed as reporter proteins. To reach higher GFP and TurboRFP expression efficiency, a double promoter P_trc-T7_ plasmid was constructed using the plasmids pET28a and pTrcHis A [37]. Briefly, the promoter P_trc_ was amplified from the template pTrcHis A using the primer pair PTrc-F and PTrc-R (Table 1), and then ligated to the plasmid pET28a. For the generation of the recombinant plasmid pET28a-P_Trc-T7_-GFP and pET28a-P_Trc-T7_-RFP, the complete sequences of the GFP and TurboRFP genes were amplified from the plasmids pEGFP and pRFP-C-RS (Clontech, Fitchburg, WI, USA) using the primer pairs GFP-F/GFP-R and RFP-F/RFP-R respectively (Table 1). The amplified complete genes were then ligated to pET28a-Ptrc_-T7_ vector using T4 DNA ligase, which had been digested by *Nco*I and *Xho*I. Subsequently, the recombinant plasmids were transformed into *Escherichia coli* S17-1λpir (AngYu Bio, Shanghai, China) to obtain S17-1λpir/pET28a-Ptrc-_T7_-GFP and S17-1λpir/pET28a-Ptrc-_T7_-RFP.

### 2.3. Bacterial Conjugation and Selection

To obtain the antibiotics required for strain fusion and screening, drug sensitivity tests were carried out on the pathogenic *V. alginolyticus* CD and probiotic *B. hwajinpoensis* RC using 6 antibiotics. Bacterial conjugation was performed followed the procedure described in a previous study [33]. Briefly, the donor strains S17-1λpir/pET28a-P_trc-T7_-GFP and S17-1λpir/pET28a-Ptrc-_T7_-RFP were grown overnight in LB medium containing 50 μg/mL *kanamycin* at 37 °C. The recipient strains (*V. alginolyticus* CD and *B. hwajinpoensis* RC) were grown overnight in 2216E medium containing 200 μg/mL of the screening antibiotic at 28 °C. Each liquid culture was then collected and washed with PBS, both strains were resuspended in 2216E medium at a concentration of 1 × 10^8^ colony forming units (CFU)/mL. Two sets of donor and recipient strains, including S17-1λpir/pET28a-P_trc-T7_-GFP × *V. alginolyticus* CD and S17-1λpir/pET28a-Ptrc-_T7_-RFP × *B. hwajinpoensis* RC, were mixed at a ratio of 3:1 (*v*/*v*) in a total volume of 2 mL. The mixtures were then centrifuged 100 revolutions per minute (RPM) to promote coupling, then the centrifuging sediments were resuspended and cultured for 6 h at 28 °C. Subsequently, the mixtures were diluted in 10^3^, 10^4^, and 10^5^ folds, and 100 μL of each dilution, and spotted onto a fresh 2216E agar plate supplemented with 50 μg/mL *kanamycin* and 200 μg/mL of the screening antibiotics. After being incubated at 28 °C for 48 h, DNA was extracted from colonies of each mixture. For the validation of successful conjugation, PCR amplification was performed with the primer pairs GFP-F/GFP-R and RFP-HF/RFP-HR, respectively. Then, the colonies grown on double antibiotics were observed using fluorescent microscope (Olympus CK40, Tokyo, Japan). Finally, the strains were further identified by sequencing of the 16S rRNA amplification with the primers 27F and 1492R.

### 2.4. Measurement of Bacterial Growth

To assess the impact of the presence of GFP and RFP genes on *V. alginolyticus* and *B. hwajinpoensis*, the growth of the GFP/RFP-tagged and wild-type strains were measured and compared as described in a previous study [38]. Briefly, a single colony of each strain was selected and cultured continuously in 200 µL of 2216E medium without antibiotics at 28 °C for 20 h. The cell density of 3 independent samples were characterized in 2 h intervals by monitoring the absorbance at 600 nm using microbial high-throughput growth curve detector (Bioscreen C, Oulu, Finland). One-way ANOVA in GraphPad Prism software (Version 9.0) was used to determine the significant differences between the control and treatment groups, with a significance level set at 5%.

### 2.5. Measurement of Bacterial Fluorescence Stability

To assess the stability of fluorescence expression in GFP-tagged *V. alginolyticus* and RFP-tagged *B. hwajinpoensis,* the fluorescence intensity in both strains was measured over a period of 12 days, with measurements taken at one-day intervals [38]. Briefly, the GFP/RFP-tagged strains were cultured overnight at 28 °C in 2216E medium supplemented with antibiotics to ensure 100% plasmid preservation and prevent contamination. A 100 µL solution was then serially diluted in a 10^5^ folds and spread onto fresh 2216E plates without antibiotics, followed by 3 independent samples that were incubated at 28 °C for 48 h. The number of fluorescent colonies and non-fluorescent colonies were determined by examining them under a fluorescence microscope and calculating their percentage relative to the total bacterial count. Then, each day, a new culture was initiated using a fluorescent colony from the previous day, and this process was continued for 12 days. 

### 2.6. Determination of Bacterial Biofilm Formation Capability

The biofilm assay was performed using the microtiter dish method described in a previous study with minor modifications [39,40]. Briefly, a single colony of GFP-tagged *V. alginolyticus* and RFP-tagged *B. hwajinpoensis* were individually selected and grown in 2216E medium without antibiotics for 12 h at 28 °C. The GFP/RFP-tagged strains were then washed 3 times with PBS and diluted in 200 µL of PBS. The bacterial suspension was added to 96-well plates and allowed to adhere for 6 h. After washing 3 times with PBS, 200 μL of 0.1% crystal violet dye was added to each well for 30 min. Next, 200 µL of 95% ethanol was added to each well and incubated on a shaker for 1 h to fully dissolve the attached crystal violet. Finally, the extent of biofilm formation was quantified by measuring the absorbance at 570 nm using Varioskan^®^ Flash enzyme markers (Thermo Scientific, Waltham, MA, USA).

### 2.7. Determination of Bacterial In Vitro Adhesion Capability

The adhesion efficiency of bacteria to slides was evaluated by bacterial cell counts [40,41]. Briefly, the fluorescent strain and wild-type were separately incubated until reaching an OD_600_ of approximately 0.5. The bacterial cultures were centrifuged and washed with PBS 3 times. Then 100 mL of each strain was placed onto sterile slides, and incubated for 6 h to achieve full adherence. The excess liquid was removed, and washed with PBS for 10 min to remove non-adhered cells. Adhered cells were eluted using an elution solution and diluted 10^3^-fold, plated on 2216E plates and incubated at 28 °C for 48 h. The adhesion capability was determined by counting the number of colonies appearing on the plates.

### 2.8. Colonization Process and Probiotic Effects

The colonization and infection process of GFP-tagged *V. alginolyticus* CD*_gfp_* and RFP-tagged *B. hwajinpoensis* RC*_rfp_* was assessed using the challenge method described in a previous study with minor modifications [42]. Briefly, a size of 60–80 μm *C. gigas* D-shaped larvae were placed on 12-well plates of containing sterile seawater at a density of 15–20 larvae per well. RFP-tagged *B. hwajinpoensis* RC*_rfp_* were inoculated in 12-well plates at a final concentration of 1 × 10^5^ CFU/mL. 6 h later, GFP-tagged and wild-type strains of *V. alginolyticus* were inoculated at a final concentration of 1 × 10^3^ CFU/mL. D-shaped larvae inoculated only with wild-type *V. alginolyticus* were used as the positive control, while uninoculated D-shaped larvae served as the negative control. The D-shaped larvae were incubated at 25 °C for 48 h. The larvae morbidity and integrity were examined using an inverted microscope (Olympus CK40, Shinjuku, Japan). Bacterial infection of the D-shaped larvae was observed and photographed using epifluorescence microscopy (40×) every 6 h, and larvae were considered dead if there was no movement. Fluorescence images showing the coexistence of GFP and RFP were acquired using the Image J software (https://imagej.nih.gov/). The enumeration of Vibrios in the seawater was performed using TCBS (Thiosulfate Citrate Bile Salts Sucrose) agar culture plates.

## 3. Results

### 3.1. Construction of Fluorescent Strains

To construct the GFP-tagged *V. alginolyticus* CD*_gfp_* and RFP-tagged *B. hwajinpoensis* RC*_rfp_*, an antibiotic marker of both strains was selected. The sensitivity of both strains to six antibiotics is presented in Table 2. The *V. alginolyticus* CD and probiotic *B. hwajinpoensis* RC exhibited resistance to *ampicillin* and *ciprofloxacin*, respectively, while both were sensitive to *kanamycin.* Therefore, *ampicillin* and *ciprofloxacin* were selected as the screening antibiotics. Then an exogenous DNA fragment containing Ptrc-GFP and Ptrc-RFP was successfully introduced to S17-1λpir, respectively, using the plasmid pET28a with *kanamycin* resistance as a carrier vector. Finally, the GFP-tagged *V. alginolyticus* CD*_gfp_* and RFP-tagged *B. hwajinpoensis* RC*_rfp_* were successfully constructed by the conjugation of S17-1λpir containing GFP and RFP genes and respective recipient strains. 

**Figure 1:**
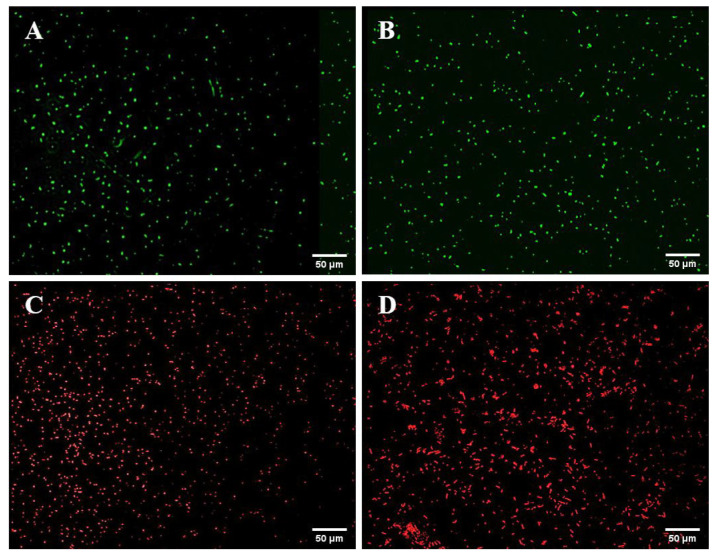
Observation of GFP-tagged and RFP-tagged strains using the fluorescence microscope: (**A**) S17-1λpir/pET28a-Ptrc-_T7_-GFP; (**B**) GFP-tagged *V. alginolyticus* CD*_gfp_*; (**C**) S17-1λpir/pET28a-Ptrc-_T7_-RFP; (**D**) RFP-tagged *B. hwajinpoensis* RC*_rfp_*.

**Figure 2:**
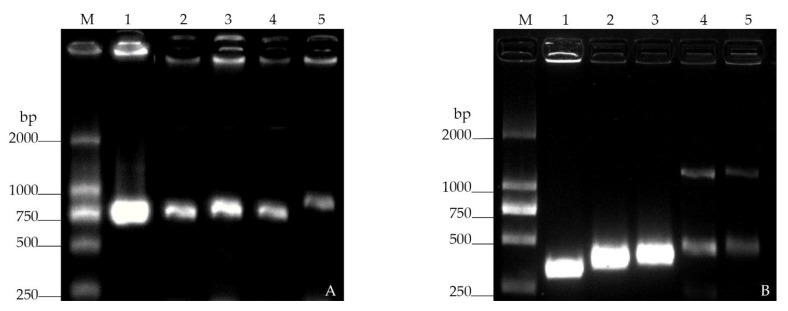
Gel electrophoresis bands of the fluorescent bacteria: (**A**) GFP-tagged strains. 1: S17-1λpir/pET28a-Ptrc-_T7_-GFP; 2~5: GFP-tagged *V. alginolyticus* CD*_gfp_*; (**B**) RFP-tagged strains. 1–3: S17-1λpir/pET28a-Ptrc-_T7_-RFP; 4~5: RFP-tagged *B. hwajinpoensis* RC*_rfp_*.

Strong fluorescence was observed under fluorescence microscopy in the GFP-tagged *V. alginolyticus* CD*_gfp_*, RFP-tagged *B. hwajinpoensis* RC*_rfp_*, and S17-1λpir/pET28a-Ptrc-_T7_-GFP/RFP (Figure 1). Furthermore, the colony PCR products displayed discernible bands on the gel electrophoresis. Nevertheless, the intensities of these gel electrophoresis bands of the recipient cells were comparatively lower than those of the donor cells (Figure 2). Through the amplification of the colonies using 16s rRNA primers, it was confirmed that both strains were the target strains, and no contamination was detected.

### 3.2. Growth Capability and Fluorescence Stability of the Fluorescent Strains

**Figure 3:**
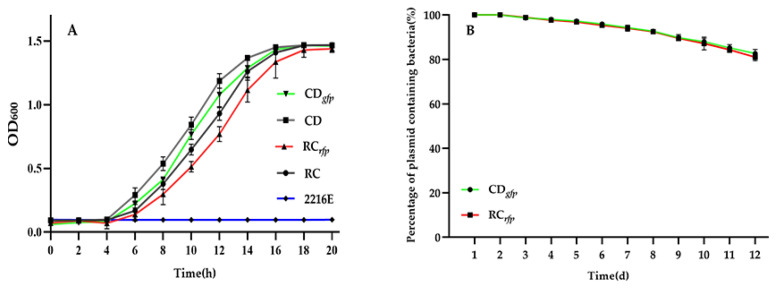
Evaluation of the biological functions of the fluorescent bacteria: (**A**) Growth curve of the wild-type strain and fluorescent strain at 28 °C in 2216E medium; (**B**) Plasmid retention in bacterial strains over time in 2216E medium. Data are the means of three independent experiments and are presented as the means ± SD.

The growth curves of the fluorescent *V. alginolyticus* CD*_gfp_*, *B. hwajinpoensis* RC*_rfp_*, and their respective wild-types were measured under the same culture conditions. Both the tagged strains and wild-types reached the exponential growth period at about 4 h post cultivation. The GFP- and RFP-tagged strains showed a relative low OD_600_ compared to their counterparts. After 20 h of growth, all cultures eventually reached the same biomass (Figure 3A). Although the OD_600_ values of FP-tagged strains were slightly lower than those of the wild-type at some time points, no significant differences were detected by ANOVA analysis. The results indicated that there was no significant difference in the growth capability of the GFP/RFP-tagged strains and respective wild-types at 28 °C.

Our GFP/RFP-tagged strains will be utilized to track the bacteria within the oyster larvae. In order to visualize them throughout the infection process, it is essential for the GFP and RFP encoding plasmids to retain stable under nonselective growth conditions. As depicted in Figure 3B, the GFP and RFP genes demonstrated efficient expression in *V. alginolyticus* CD*_gfp_* and *B. hwajinpoensis* RC*_rfp_* over a continuous period of 12 days. Furthermore, 82% of the fluorescent bacteria maintained a high fluorescence level at day 12. These findings indicate that the exogenous GFP and RFP genes were able to persist through multiple passages in a medium without antibiotics. These results showed that the constructed GFP/RFP-tagged strains could meet the requirement for observing bacterial colonization in the larvae over a 7-day period.

### 3.3. Biofilm Formation and In Vitro Adhesion of the Fluorescent Strains

**Figure 4:**
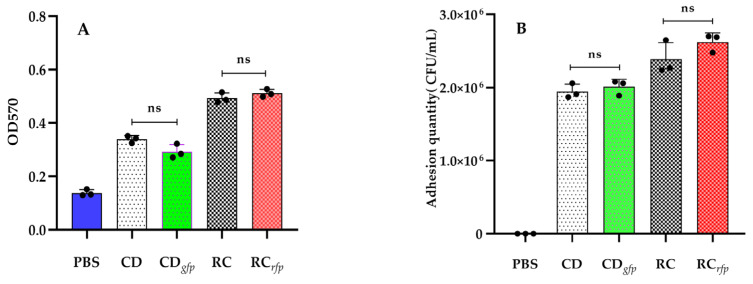
Indicators of colonization capability of the fluorescent bacteria: (**A**) Biofilm formation of blank control (PBS), wild-type (CD, RC) and fluorescent strains (CD*_gfp_*, RC*_rfp_*); (**B**) The adhesion of blank control (PBS), wild-type (CD, RC) and fluorescent strains (CD*_gfp_*, RC*_rfp_*) to slide demonstrated by fluorescence colony counting. Data are the means of three independent experiments (•) and are presented as the means ± SD. ns: no significant difference (*p* > 0.05).

Crystalline violet staining was conducted to assess biofilm formation in both fluorescent bacteria and wild-type strains. OD_570_ values of the blank group (PBS) and wild-type strains (CD and RC) were 0.1379 ± 0.0122, 0.3395 ± 0.0132, and 0.4932 ± 0.0192, respectively, which indicated that both of the wild-type strains were capable of forming biofilms. For *V. alginolyticus* CD*_gfp_* and *B. hwajinpoensis* RC*_rfp_*, the OD_570_ values were determined to be 0.2924 ± 0.0268 and 0.5112 ± 0.0145, respectively. The number of biofilms formed by the fluorescent strains did not significantly differ from those formed by the wild-type strains (Figure 4A). These results suggested that the introduction of GFP and RFP genes did not have a substantial impact on biofilm formation.

To evaluate the adhesion efficiency of the fluorescent strains to slides, plate colony counting and fluorescence microscopy were employed in this study. The wild-type strains (CD and RC) achieved adhesion levels of 1.95 ± 0.10 × 10^6^ CFU/mL and 2.39 ± 0.23 × 10^6^ CFU/mL, whereas the fluorescent strains (CD*_gfp_* and RC*_rfp_*) achieved levels of 2.01 ± 0.10 × 10^5^ CFU/mL and 2.62 ± 0.12 × 10^5^ CFU/mL, respectively. The results indicated that the *B. hwajinpoensis* exhibited relatively higher adhesion to the slides compared to the *V. alginolyticus*. The fluorescent strains (CD*_gfp_* and RC*_rfp_*) and corresponding wild-type strains (CD and RC) showed no significant difference in the adhesion ability (Figure 4B).

### 3.4. Colonisation Process and Effect on Survival of Fluorescently Tagged Strains in C. gigas D-Sharped Larvae

**Figure 5:**
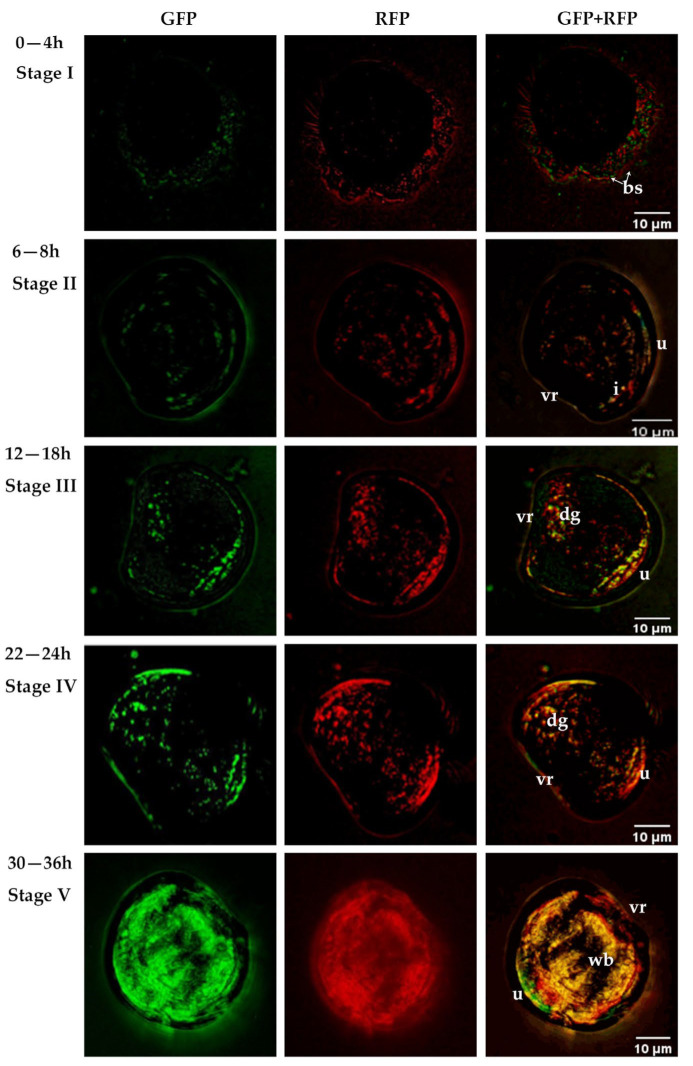
Colonization process of fluorescently tagged bacterial in D-shaped larvae under a fluorescence microscope. bs: bacterial swarming; dg: digestive gland; i, intestine; u: umbo; vr: ventral region.

The colonization process of *V. alginolyticus* CD*_gfp_* and probiotic *B. hwajinpoensis* RC*_rfp_* in *C. gigas* D-sharped larvae at 25 °C was observed. The larvae were healthy at the start of infection (0 h). By 4 h of infection, fluorescent bacteria start to adhere and accumulate around the larvae (Figure 5 Stage I). Between 6–8 h of infection, the bacteria entered the digestive organs of the larvae, such as the stomach and intestine, through the esophagus and established colonization in these areas (Figure 5 Stage II). From 12–18 h of infection, the bacteria spread from the dorsal to the abdomen (Figure 5 Stage III). By 22–24 h of infection, the fluorescence intensity in the larval abdomen increased significantly and some bacteria fully colonized the larval (Figure 5 Stage IV). The peak of bacterial colonization was reached at 30–36 h of infection (Figure 5 Stage V).

**Figure 6:**
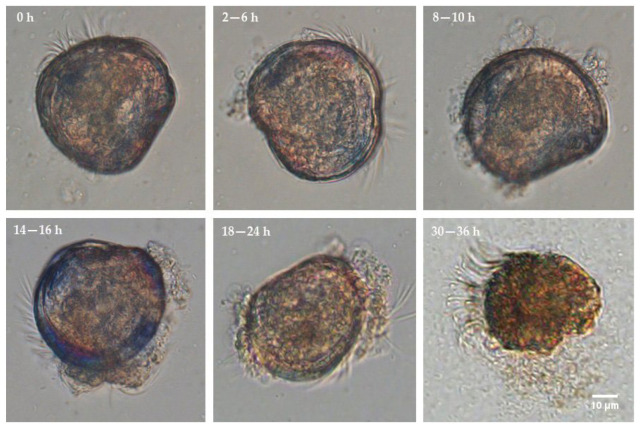
Main symptoms of pathogenic activity of *V. alginolyticus CD_gfp_* on experimentally infected *C. gigas* D-sharped larvae.

**Figure 7:**
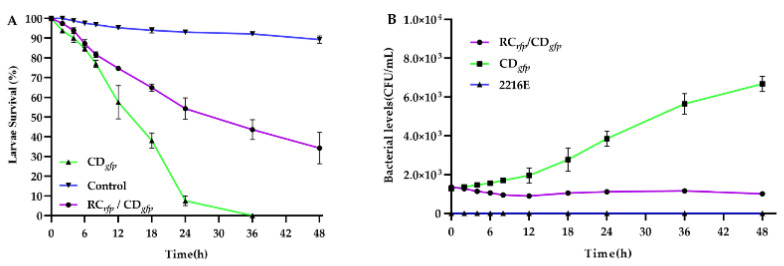
Pathogenic and probiotic effects of *B. hwajinpoensis* RC*_rfp_* and *V. alginolyticus* CD*_gfp_* on D-sharped larvae: (**A**) Survival of *C. gigas* D-sharped larvae in the challenged and control groups; (**B**) Vibrio levels in seawater during larval challenge experiments. Data are the means of three independent experiments and are presented as the means ± SD.

After administration of *V. alginolyticus* CD*_gfp_*, the larvae did not display any noticeable signs of illness until the early stage II (6 h) (Figure 6). The concentration of bacteria around the larvae increased steadily during the late stage II and early stage III (8–10 h). At this point, the initial symptoms became apparent, characterized by reduced mobility. By 18–24 h post-infection (late stage III and stage IV), the structure changes were evident in organs such as villi, cilia, and the stomach interior. Larvae mortalities were also initiated at 8 h post-infection, and reached 92.48% and 100% at about 24 h and 36 h post-infection, respectively (Figure 7A). Bacterial colonization, disrupted, and disintegrated larval soft tissues could be observed frequently in and around dying and dead larvae in this period (Figure 6). The larvae treated by both *B. hwajinpoensis* RC*_rfp_* and *V. alginolyticus* CD*_gfp_* displayed mortality rates of 45.71%, 56.39% and 65.76% at 24 h, 36 h and 48 h, respectively. These mortality rates were significantly lower than those observed in the *V. alginolyticus* CD*_gfp_* treatment group (Figure 7A). The negative control group exhibited no significant symptoms, with average mortality rates of 6.89%, 7.81% and 10.71% at 24 h, 36 h and 48 h, respectively. 

Larvae inoculated with *V. alginolyticus* exhibited an increasing levels of *V. alginolyticus* over 48 h. At 48 h, the average concentration of *V. alginolyticus* in the water was recorded as 0.67 × 10^4^ CFU/mL. The *V. alginolyticus* level at 48 h was 5.48 times higher than that of 0 h (Figure 7B). On the other hand, the levels of *V. alginolyticus* were relatively stable over 48 h, when *B. hwajinpoensis* RC was present along with *V. alginolyticus*. These results indicated that probiotics exhibited an inhibitory effect on the growth of *V. alginolyticus* in larvae (Figure 7B).

## 4. Discussion

The Pacific oyster is one of the most important cultured marine species, with a high value and strong market demand around the world. It is estimated that the production of triploid Pacific oyster seeds increased from about 2.3 billion in 2018 [43], to about 60 billion in 2022 in China (Zhang, personal communication). Hatchery production of the triploid oyster seed is crucial for ensuring a constant and sufficient supply of juveniles to support the oyster industry. Changes in environmental conditions and disease outbreaks caused by pathogenic *Vibrio* spp. have constituted the main threats to the hatchery production process [13]. In an effort to maintain a healthy cultivation system, bivalve hatcheries try their best to maintain optimum water quality by controlling larval culture density, the use of water treatment systems and renewing the waters at a suitable frequency, according to experience [44]. Filtration, combined with UV-sterilization or ozone, has been recommended as the preferred method to sterilize all or part of the seawater before use [45]. However, the traditional breeding facilities and technologies for bivalve hatcheries were still widely employed for production in China [46]. These breeding methods were developed in the 1980s, which were characterized by poor facilities and management practices [46]. Before being introduced to the hatchery, the seawater from the open sea nearby was always only treated by flowing through once or twice of sand filter tanks. Vibrios including the potential pathogenic *V. alginolyticus* and *V. mediterranei,* are ubiquitous in marine and estuarine aquatic systems [6,9,47]. It is impossible to eliminate the potential pathogenic microorganisms from the bivalve hatcheries [7,9,47]. Moreover, there are always no biosecurity protocols, hygiene plans, or risk-based surveillance schemes for these family-style bivalve hatcheries in China [46]. The diseased larvae and hatchery effluents are always discharged directly into drains connecting to the open sea. It is common to find dozens of bivalve hatcheries densely distributed along the coastline, which will definitely exacerbate the spread of infectious diseases. All these factors combined together make pathogenic bacteria a major problem for hatchery production, and cause severe diseases and high mortality, which seriously affect hatchery production and cause significant economic loss. Antimicrobial agents have been employed for the control and treatment of bacterial disease in mollusk hatcheries. However, the use of antibiotics may lead to the rapid development of resistant pathogen populations, the elimination of beneficial organisms and the emergence of other microbial pathogens [15,16].

For the distinct disadvantages and regulation limitations of antibiotic usage, probiotics were widely used in aquaculture and bivalve hatcheries as an alternative strategy to control infection and regulate water quality [22,24,48,49,50]. Most of these studies have shown that probiotics can significantly improve the growth and survival of bivalve larvae. Daily addition of a mixture of S4 and RI (10^4^ CFU/mL) could result in a significant decrease in the levels of total Vibrios in water and tank surfaces [51]. Use of the probiotics *Phaeobacter gallaeciensis*, *Alteromonas macleodii* 0444 and *Neptunomonas* sp. 0536 (10^4^–10^5^ CFU/mL) could increase the survival of *Pecten maximus* larvae by 50.2% [52]. As reviewed previously, many studies have shown that probiotics could act as pathogen inhibitors, ecological modifiers, and host growth promoters in bivalve hatcheries, with no negative effect on larval growth and survival [22,24,48]. Previous studies also showed that the mechanism of action of probiotics is stage- and species-specific, with different responses in different hosts [53,54]. In this study, the probiotic *B. hwajinpoensis* RC was derived from healthy adult oyster gut tissue, whose biological functions have been investigated in previous studies and does not possess relevant virulence gene [55]. Usage of probiotics of 10^5^ CFU/mL significantly inhibited the growth of pathogenic *V. alginolyticus* and reduced the mortality of D-sharped larvae.

Initial studies on the colonization of pathogenic bacteria in larvae of different bivalves such as oysters, scallops and mussels were carried out mainly by microscopic histological observation [15,56,57]. However, this method does not allow the main pathogens to be distinguished from conventional microorganisms in larval tissues. The use of fluorescent labelling of bacteria can be used in pathological studies to visualize the distribution, content and progression of infection within an organism [37]. Dubert et al. (2016) resolved the occurrence and development of vibriosis in *Ruditapes philippinarum* larvae by GFP labelling of pathogenic Vibrios [34]. Wang et al. (2021) used GFP to label *Vibrio splendens* ME9 and *Vibrio eelis* NB10, combined with histopathological analysis and ultrastructural observation, to resolve the pathogenesis of Vibrios in blue mussel larvae within 24 h [36]. Cabello et al. (2005) reported that about 85% of GFP-labelled *Vibrio Parahaemolyticus* in seawater adhered to, entered, and colonized the oyster through the gills [58]. GFP was also proved to be an ideal visualization tool to clearly study the localization of bacteria in different organs, entry routes, and the extent of bacterial infection of different tissues (gill epithelial cells and hemolymph sinus) in dying abalone [59]. However, some studies have also suggested that the introduction of fluorescent proteins into bacteria may adversely affect the growth, virulence, or other physiological functions of certain pathogenic bacteria [38,60]. Therefore, it is recommended that differences between the physiological functions of wild-type and GFP-labelled strains should be compared firstly to confirm the reliability of results obtained from GFP-labelled strains [61]. Biofilm formation is crucial for both pathogenic and probiotic bacteria to complete the process of adhesion and colonization [39]. For pathogenic microorganisms, biofilm formation not only plays an important role during invasion, but also protects them from host immune system [62]. On the other hand, biofilm-forming probiotics have significant advantages in combating pathogens, resistance, and immunomodulation [25]. Moreover, biofilms generated by specific microbial species play an important role during the settlement of oyster larvae [63,64]. Larval settlement always increased with biofilm age and mass [63]. In the present study, GFP and RFP were employed to label the pathogenic *V. alginolyticus* CD and probiotic *B. hwajinpoensis* RC strain, respectively, with the aim to distinguishing the two strains when they attached and entered the oyster larvae. No significant differences were found in capabilities of growth, biofilm formation and in vitro adhesion between the fluorescence-labelled and wild strains in the present study. Additionally, previous studies in animal models (Drosophila) have demonstrated that oxygen levels in the host organism could impact the in vivo distribution of GFP and RFP [65]. While oysters have an open circulatory system consisting of the heart, arteries, and veins connected to blood sinuses throughout the connective tissues [66]. The blood is in direct contact with the cell membranes of oyster tissues and the hemocytes can massively infiltrate the tissues of the oyster visceral mass. According to our knowledge, there is no report about oxygen disequilibrium in oyster tissues. It is difficult to estimate the influence of oxygen levels on the fluorescence intensity due to the scant information available.

Since the formation of chromophores for both GFP and RFP depends on the presence of oxygen, hyper- and hypoxic- environments in the host organism have shown to reduce both fluorescence intensity and expression levels of these proteins. However, the findings of this study have not been documented in bivalve mollusks. Furthermore, none of the previous studies on GFP- and RFP-labeled bacteria colonization and distribution in aquatic animals have mentioned the potential detrimental impact of oxygen levels on GFP and RFP fluorescence. Therefore, the current study investigated the interactions between pathogenic and probiotic bacteria through an analysis of their distribution in oyster larvae, without taking the issue of oxygen content into account. Furthermore, we hypothesized that the maturation of GFP and RFP in bacteria relies on internal oxygen supply. In an oyster body, if certain areas lack sufficient oxygen to support bacteria growth, it is possible that bacterial distribution and colonization may be limited. In this study, clear visualization of the colonization process with fluorescence labelling showed that the pathogenic *V. alginolyticus* CD and probiotic *B. hwajinpoensis* RC strains adopted nearly the same routes during the entering and colonization in the oyster larvae. The distribution of bacteria in various sections of the oyster larvae was visible at different time intervals. Consequently, any fluorescent proteins affected by inadequate oxygen levels and hence, not fully matured whether in this study can be disregarded. In future studies, we will consider examining whether the expression of fluorescent proteins increases the aerobic burden on bacteria.

Studies have shown that ciliated bands on the membrane of bivalve larvae are capable of enriching bacteria and transferring them to the oral cavity [67]. The enriched bacteria subsequently pass through the esophagus to the digestive organs such as the stomach and intestine, where it subsequently proliferates rapidly and then spreads to the body cavity, and eventually colonizes the larvae completely [67]. In the present study, two fluorescently labelled bacteria were used for the first time to monitor their colonization process in *C. gigas* D-sharped larvae, and the results support the conclusions of Elston and Leibovitz (1980). Furthermore, clear visualization of the colonization process with fluorescence labelling showed that the pathogenic *V. alginolyticus* CD and probiotic *B. hwajinpoensis* RC strains adopted nearly the same routes during the entering and colonization in the oyster larvae. These results indicated that *B. hwajinpoensis* RC could execute its’ probiotic effects by competing for adhering and colonization sites with *V. alginolyticus.* Studies have noted that probiotics repel pathogens when they are present before the pathogen, but not when both are added at the same time [68]. In this study, the pathogenic *V. alginolyticus* CD was added to the bioassay system 6 h later than the probiotic *B. hwajinpoensis* RC. The bacterial level of *V. alginolyticus* CD in the probiotic treated group were significantly higher than the control group (challenged with *V. alginolyticus* CD). The survival rate of oyster larvae in the treated group was significantly higher than that in the control group. 

## 5. Conclusions

In summary, the GFP and RFP genes were for the first time used to label pathogenic *V. alginolyticus* and *B. amyloliquefaciens*, respectively. Our results demonstrated that the introduction of the exogenous genes did not significantly affect the physiological functions of the bacteria. Labelling the strains with different fluorescent proteins made it possible to visualize the colonization process of both pathogenic and probiotic bacteria in *C. gigas* larvae. It was observed that a similar route was employed to enter and colonize the oyster larvae by *V. alginolyticus* and *B. amyloliquefaciens*. It was concluded that competing with pathogens for binding and spreading sites were one of the mechanisms of *B. hwajinpoensis* to provide the probiotic effects to oyster larvae. The results indicated that the employment of fluorescence-tagged pathogenic and probiotic strains simultaneously provides us a tool to understand the potential mechanisms of the probiotics.

## Figures and Tables

**Table 1:** All primer sequences and reaction conditions used in this study.

Primer	Primer Sequence (5′-3′) *	Enzyme Site	Size (bp)	Annealing Temperature (°C)
27-F	AGAGTTTGATCCTGGCTCAG	none	1517	55
1492-R	TACGACTTAACCCCAATCGC			
PTrc-F	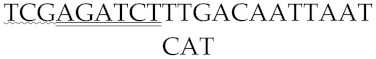	Bg*l* I	48	56
PTrc-R	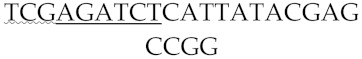			
GFP-F	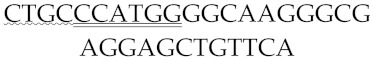	Nco I	738	60
GFP-R	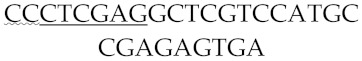	Xho I		
RFP-F	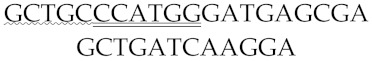	Nco I	715	58
RFP-R	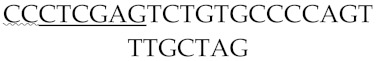	Xho I		
RFP-HF	CGACTTCTTTAAGCAGTCCTTCC	none	336	55
RFP-HR	ATCTTGAGGTTCTTAGCGGGTTT			

* Nucleotides wavy underlined represent guard base; Nucleotides double underlined represent restriction enzyme site; Nucleotides underlined represent reverse complement.

**Table 2:** Results of drug sensitivity test of recipient strain and donor strain.

Antibiotics	Strains		
	*V. alginolyticus* CD	*B. hwajinpoensis* RC	*E. coli* S17-1λpir/pET28a-P_trc-T7_-GFP/RFP
*Penicillin*	+	−	++
*Ampicillin*	−	+++	+++
*Kanamycin*	+++	++	−
*Gentamicin*	+++	++++	+++
*Erythromycin*	+++	+++	−
*Ciprofloxacin*	+++	−	+++

According to the National Committee for Clinical Laboratory Standardization (NCCLS) recommended standard inhibition circle diameter (mm): extremely sensitive (++++) > 20 mm; 15 mm ≤ highly sensitive (+++) ≤ 20 mm; 10 mm ≤ moderately sensitive (++) ≤ 14 mm; hypoallergenic (+) <10 mm; −: not sensitive.

## Data Availability

The authors confirm that the data supporting the findings of this study are available within the article.
